# SARS-COV-2 IgG and IgM Antibodies in Cancer Patients

**DOI:** 10.31557/APJCP.2021.22.8.2311

**Published:** 2021-08

**Authors:** Rujittika Mungmunpuntipantip, Viroj Wiwanitkit

**Affiliations:** 1 *Private Academic Consultant, Bangkok Thailand. *; 2 *Honorary Professor, Dr DY Patil University, Pune, India. *

**Dear Editor**

We would like to share ideas on “Evaluation of Serologic Changes of IgG and IgM Antibodies Associated with SARS-COV-2 in Cancer Patients: A Cohort Seroprevalence Study (Arab et al., 2021). Arab et al., (2021) concluded that “in the present study asymptomatic cancer patients revealed 17% seropositivity, approximately equal to the general population of the same age, sex, geographic region, and epidemic status.” We agree that there is a risk of disease transmission from cancerous patients with asymptomatic COVID-19. Nevertheless, it should discuss on the efficacy of the diagnostic test for assessment of infection. In the present report, the Pishtaz Teb SARS-CoV-2 ELISA kit is used but there is no data on diagnostic property of the test. There is extremely limited information on the Pishtaz Teb SARS-CoV-2 ELISA kit in international literature. Different types of ELISA tests targeting at different targets can result in different sensitivity and specificity (Vengesai et al., 2021; Huber et al., 2021). False positive is also a possible problem (Vengesai et al., 2021; Huber et al., 2021). For further generalization of the finding for disease control plan in any setting, it is necessary to have a validation on diagnostic property of the diagnostic test.
